# 2958. Investigating the mechanisms of persistent inflammation and the association with non-communicable diseases (NCDs), Tuberculosis (TB) recurrence, and other infections

**DOI:** 10.1093/ofid/ofad500.197

**Published:** 2023-11-27

**Authors:** Santiago Carrero Longlax, Andrew DiNardo, Alexandra Portillo, Tomoki Nishiguchi, Daniel M Musher, Ntombifuthi Ginindza, Anna Mandalakas, Clement gascua, Durbbin Mulengwa

**Affiliations:** BCM, Houston, TX; Baylor College of Medicine, Houston, Texas; Baylor College of Medicine, Houston, Texas; BCM, Houston, TX; Michael E. DeBakey VA Medical Center / Baylor College of Medicine, Houston, Texas; Ministry of Health, Mbabane, Hhohho, Swaziland; Baylor College of Medicine/Texas Childrens Hospital, Houston, Texas; National Institute for Communicable Diseases, johannesburg, Gauteng, South Africa; Baylor College of Medicine, Houston, Texas

## Abstract

**Background:**

TB continues to be a major cause of morbidity and mortality worldwide. Despite successful completion of anti-tuberculosis therapy (ATT), studies indicate that TB survivors (post-TB) have a significantly higher risk of mortality compared to the general population, primarily due to increased incidence of non-communicable diseases (NCDs; cardiovascular disease, cancer, lung disease) and recurrent infections. Furthermore, a considerable proportion of TB patients exhibit persistent inflammation even after ATT.

**Methods:**

We enrolled 325 individuals with microbiologically confirmed pulmonary TB, diagnosed between 2014 and 2020. Blood samples were obtained at baseline and 12- months (6-months after completing antimicrobial therapy) to evaluate persistent inflammation. Plasma C-reactive protein (CRP) was used to measure inflammation. Lipid peroxidation (LP) was measured by oxidized (HNE) LDL. Redox capacity was measured via the Trolox assay. Immune activation was measured by the Indoleamine 2,3 dioxygenases (IDO) enzyme by measuring the Kynurenine-Tryptophan ratio.

**Results:**

After successful TB treatment, 62% of participants exhibited persistent elevation of CRP (P< 0.001) (Figure 1a). Univariate analysis of the first 186 participants revealed no association between elevated post-TB inflammation and HIV status (Fig. 1B), gender, or age. Individuals with increased CRP levels had a lower redox capacity compared to those whose CRP normalized at the end of ATT (P< 0.001) (Figure 2). Analysis of Oxidized LDL in successfully treated TB patients demonstrated that low antioxidant capacity also had persistent LP (Figure 2b). IDO activity was increased among TB participants with high CRP, which persisted even after successful therapy in participants with high and low CRP levels (Figure 3).

Fig 1a:

**Figure:**
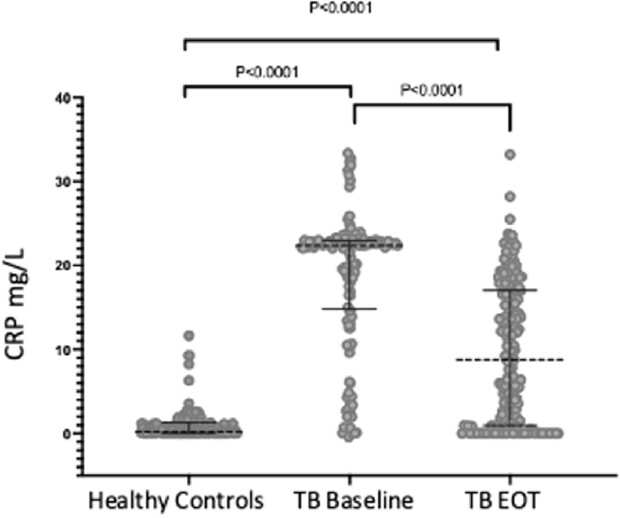

CRP was evaluated on TB participants and their asymptomatic, healthy household contacts. The median CRP of the healthy contacts was <2 mg/L, whereas the median CRP for TB participants at baseline was 21 and decreased to 8 at the end of successful therapy (EOT). Previous studies have demonstrated that CRP levels >3 mg/L are associated with increased CVD risk . 62% (111/186) of TB participants have CRP levels >3 mg//L at the end of TB therapy.

Fig 1b:

**Figure:**
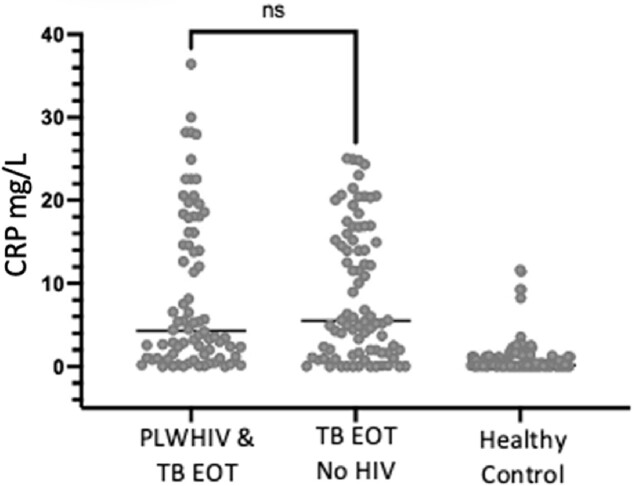

All TB participants living with HIV (PLWH) are started on ART, with >90% having a viral load <1000 at end of therapy (EOT). At EOT, TB participants without HIV do not have lower CRP levels compared to PLWHIV, preliminarily suggesting HIV is not driving the persistent inflammation.

Fig2a:

**Figure:**
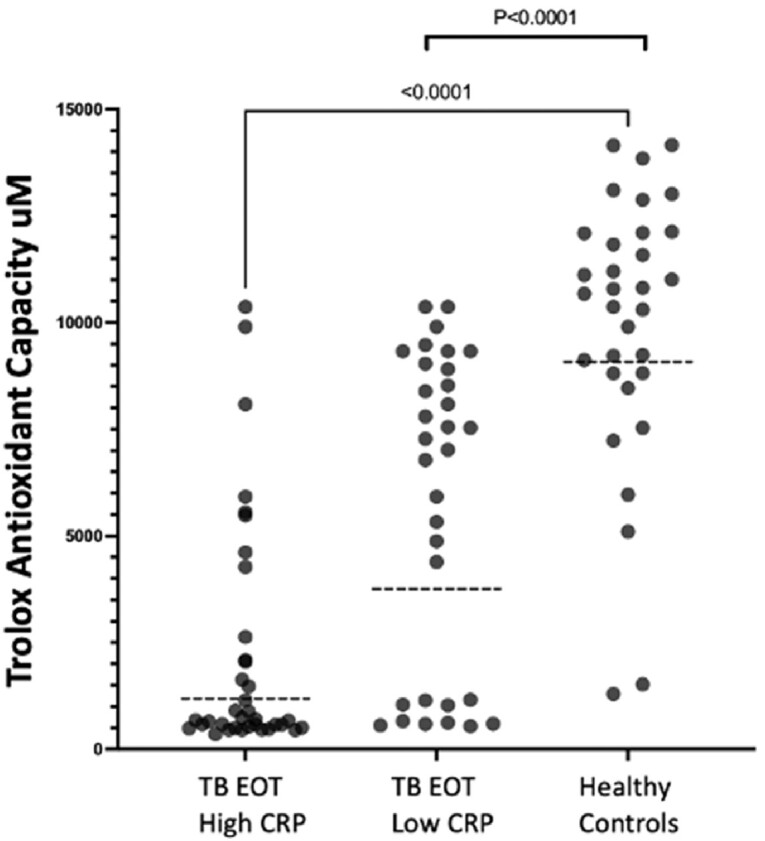

The Trolox antioxidant capacity was evaluated on TB Participants with High CRP >3mg/L (n:36) vs Low CRP <3mg/L (n:32) and Healthy Controls (n:32).

**Conclusion:**

This study shows that a substantial proportion of TB participants exhibit persistent inflammation and IDO activity even after ATT. Among TB participants who do not normalize inflammation at the end of successful therapy, there is also persistent LP and decreased redox capacity. This suggests that there is a bidirectional malignant relationship between LP and redox capacity. Potentially, this suggests that mitigating lipid peroxidation may help decrease post-TB inflammation.

Fig2b:

**Figure:**
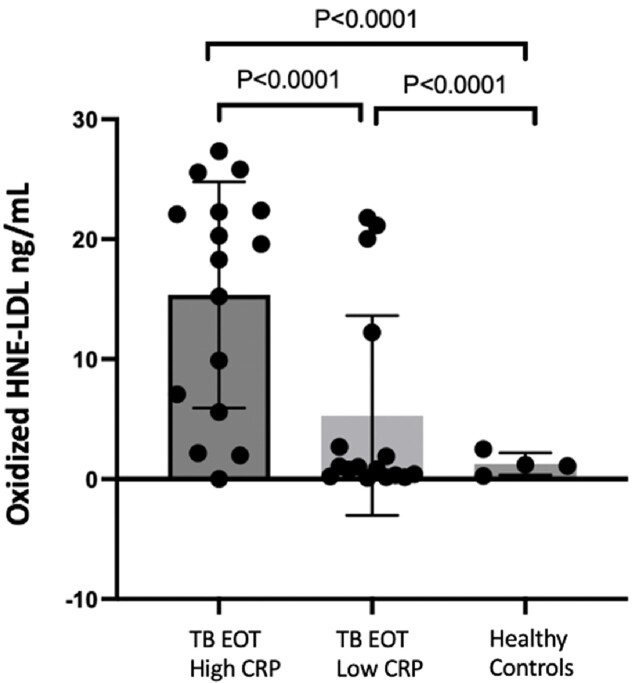

Oxidized LDL was measured in TB individuals with High levels of CRP High CRP >3mg/L (n:16) vs Low CRP <3mg/L (n:16) and Healthy Controls (n:4)

Fig3

**Figure:**
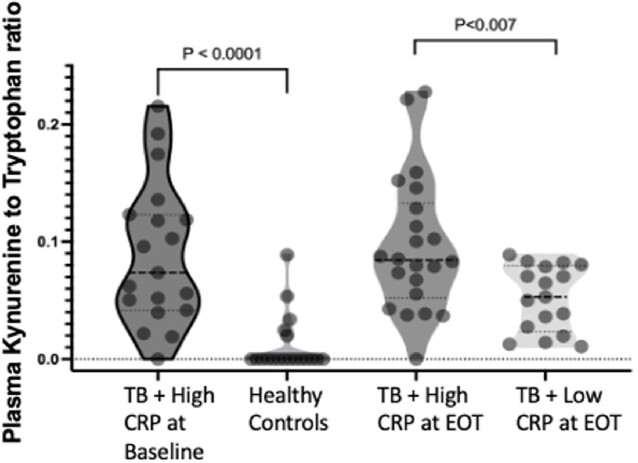

Plasma Kynurenine to Tryptophan ratio was evaluated in TB individuals with persistent augmented levels of CRP (n:19) at baseline and Heathy Controls. At the end of Therapy the KYN:TRYP was evaluated in TB individuals with high (n:22) and Low CRP levels (n:17)

**Disclosures:**

**All Authors**: No reported disclosures

